# Live Imaging of Adult Neural Stem Cells in Rodents

**DOI:** 10.3389/fnins.2016.00078

**Published:** 2016-03-07

**Authors:** Felipe Ortega, Marcos R. Costa

**Affiliations:** ^1^Biochemistry and Molecular Biology Department, Faculty of Veterinary Medicine, Complutense UniversityMadrid, Spain; ^2^Brain Institute, Federal University of Rio Grande do NorteNatal, Brazil

**Keywords:** live imaging, aNSCs, neurogenic niches, timelapse videomicroscopy, lineage tracing

## Abstract

The generation of cells of the neural lineage within the brain is not restricted to early development. New neurons, oligodendrocytes, and astrocytes are produced in the adult brain throughout the entire murine life. However, despite the extensive research performed in the field of adult neurogenesis during the past years, fundamental questions regarding the cell biology of adult neural stem cells (aNSCs) remain to be uncovered. For instance, it is crucial to elucidate whether a single aNSC is capable of differentiating into all three different macroglial cell types *in vivo* or these distinct progenies constitute entirely separate lineages. Similarly, the cell cycle length, the time and mode of division (symmetric vs. asymmetric) that these cells undergo within their lineage progression are interesting questions under current investigation. In this sense, live imaging constitutes a valuable ally in the search of reliable answers to the previous questions. In spite of the current limitations of technology new approaches are being developed and outstanding amount of knowledge is being piled up providing interesting insights in the behavior of aNSCs. Here, we will review the state of the art of live imaging as well as the alternative models that currently offer new answers to critical questions.

## Introduction

### Current knowledge of the cytoarchitecture of the adult neurogenic niches; subependymal zone and dentate gyrus

**Figure 1:**
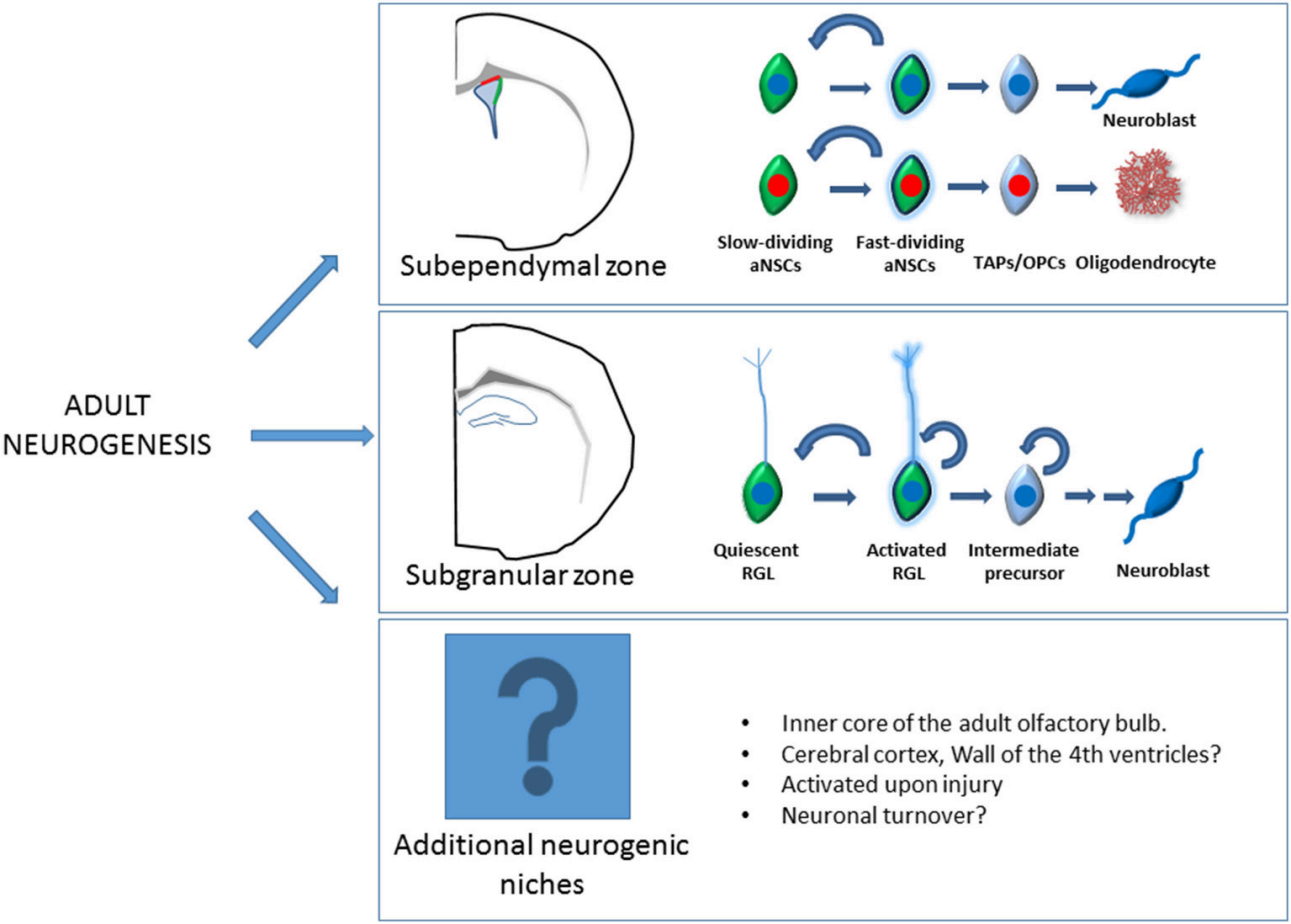
**Schematic representation of the adult neurogenesis**. Here there are depicted the two main adult neurogenic niches, the subependymal zone in the lateral wall of the lateral ventricle and the subgranular zone in the hippocampus. Live imaging experiments have shown than within the SEZ, neurogenic, and oligodendrogliogenic lineage follows a similar pattern of lineage progression but constitutes independent lineages. Slow dividing astroglia (quiescent type B cells) give rise to fast dividing astroglia (activated type B cells) that subsequently generates Transit amplifying progenitors (TAPs) and finally neuroblast or oligodendrocytes. In the SGZ, quiescent radial glia like (RGL) progenitors become activated giving rise to intermediate progenitors and neuroblast that undergoes a complex process of maturation. Additional neurogenic niches like the olfactory bulb or the cerebral cortex have also been reported. The existence of undiscovered neurogenic niches should not be discarded. Several regions of the adult brain reactivate “dormant” aNSCs through signaling pathways released upon injury. Likewise, contribution of these quiescent aNSCs to the periodical turnover of neural populations still remains to be demonstrated.

Stem Cells are defined by their ability to self-renew, giving rise to new stem cells, and their capacity to generate diverse specialized cell types. In the adult nervous system, the term neural stem cells (NSCs) refers to cells that maintain the capacity to self-renew and generate neurons and macroglial cells both *in vitro* (Reynolds and Weiss, 1996; Costa et al., 2011) and *in vivo* (Lois and Alvarez-Buylla, 1993; Gould and Cameron, 1996; Kempermann et al., 1997; Menn et al., 2006; Sohn et al., 2015). Adult neural stem cells (aNSCs) continuously generate neurons oligodendrocytes and astrocytes in discrete niches in the brain, although it is unclear whether multipotent or unipotent aNSCs contribute all these different lineages. Historically, the adult neurogenesis has been associated, under physiological conditions, to two specific neurogenic niches: the subependymal zone (SEZ) in the lateral wall of the lateral ventricle, and the subgranular zone (SGZ) of the dentate gyrus in the hippocampus reviewed by Gage (2000) and Kriegstein and Alvarez-Buylla (2009). However, the presence of aNSCs in alternative domains of the adult brain should not be discarded. Indeed, multipotent progenitors have been isolated from the postnatal mouse cerebral cortex (Marmur et al., 1998; Belachew et al., 2003; Seaberg et al., 2005; Costa et al., 2007) or adult mouse cerebral cortex after traumatic and ischemic lesion (Buffo et al., 2008; Sirko et al., 2013). Another interesting adult domain described to contain NSCs is the inner core of the olfactory bulb (OB) of both rodents and humans. Populations of NSCs expressing GFAP, Nestin, Sox2, and RC2 are located within the adult OB giving rise to neurons *in vivo*. Likewise they can be expanded *in vitro* as neurospheres, giving rise to astrocytes, oligodendrocytes and neurons. (Pagano et al., 2000; Gritti et al., 2002; Liu and Martin, 2003; Giachino and Taylor, 2009; Vergano-Vera et al., 2009; Moreno-Estelles et al., 2012). The same is applied for human temporal and frontal cortex, amygdala and hippocampus after resection due to a drug-resistant epilepsy, dysplasia, trauma, or brain edema (Arsenijevic et al., 2001). More recent evidence indicate that lesions may activate those “dormant” aNSCs through release of signaling molecules such as vascular endothelial growth factor (VEGF), basic fibroblast growth factor (bGFG), and sonic hedgehog (SHH; Sirko et al., 2013; Luo et al., 2015). Contribution of these quiescent aNSCs to a possible periodical and so far unnoticed turnover of their associated neuronal populations remains to be demonstrated Figure 1.

Focusing on the two main neurogenic niches of the adult brain, the SEZ harbors a population of aNSCs, known as type B cells, located beneath the ependymal cell layer of the lateral ventricles (Doetsch et al., 1999a,b). Type B has been proposed to share a common lineage with embryonic radial glia (RG) (Merkle et al., 2004). However, whether type B cells constitute the endpoint or RG lineage progression or whether the divergence arrives earlier is a matter of debate. Recent research went into detail about the relationship between RG and aNSCs (Fuentealba et al., 2015; Furutachi et al., 2015). Interestingly, evidences suggest that RG give rise to a subset of progenitors named pre-B1 cells at early stages of embryonic neural development (E 13.5-E 15.5). Pre-B1 cells remain quiescent until adulthood when they become reactivated. Furutachi and colleagues, points to the slowing down of the cell cycle of a subset of RG as the responsible for the origin to the adult pool of NSCs. Moreover, the cyclin-dependent kinase inhibitor p57 is proposed as the key regulator of the RG cell cycle. Selective deletion of p57 blocked the deceleration of the cell cycle, impairing the emergence of adult NSCs. These evidences are also supported by long-term *in vivo* experiments that described, using the STARTRACK tracing system, the presence of adult progenitors with embryonic origins (E-14), remaining as small, round and quiescent cells on the SEZ (Garcia-Marques and Lopez-Mascaraque, 2013). Type B cells are characterized by the expression of astroglial proteins such as the glial-fibrillary acidic protein (GFAP) and glutamate-aspartate transporter (GLAST; Doetsch et al., 1997; Platel et al., 2009). These cells contact the lateral ventricle through a single primary cilia located in the apical zone where they form junctional complexes among themselves (Mirzadeh et al., 2008). They also exhibit long basal processes that are closely associated with blood vessels, suggesting that vascular derived signals could play a role of the in the regulation of aNSC behavior (Mirzadeh et al., 2008; Shen et al., 2008; Tavazoie et al., 2008; Calvo et al., 2011). Regarding the cell cycle, type B cells are relatively quiescent (Doetsch et al., 1999a) but become activated giving rise to activated astroglia and type C cells or transit amplifying progenitors (TAPs; Pastrana et al., 2009; Costa et al., 2011). TAPs are more active in proliferation, undergoing several rounds of amplifying divisions (Costa et al., 2011; Ponti et al., 2013) before giving rise to neuroblasts or Type A cells. Subsequently, neuroblasts form a chain migrating along the rostral migratory stream toward the olfactory bulb (Lois et al., 1996). After reaching the olfactory bulb neuroblasts differentiate into different subtypes of neurons (Lledo et al., 2006; Fiorelli et al., 2015). The majority becomes GABAergic granule neurons and a minority becomes GABAergic periglomerular neurons. In addition, a small percentage of neuroblasts, generated in the dorsal wall of the SEZ, turn into short-axon glutamatergic, or GABAergic juxtaglomerular neurons (Kosaka and Kosaka, 2008; Brill et al., 2009; Kiyokage et al., 2010) Moreover, at least four subtypes of OB interneurons are generated at the anterior ventral SEZ, arising from microdomains that correlate with the expression domains of the Nkx6.2 and Zic family of transcription factors (Merkle et al., 2014).

Specific molecular markers to identify type B cells population have not been available for a long time. However recent publications employed interesting combinations of markers to differentiate between quiescent and activated type B cells, allowing for their isolation. Vascular cell adhesion molecule 1 (V-CAM1) has been recently described as a marker for quiescent type B cells (Kokovay et al., 2012). Likewise, combination of GFAP, prominin1 and the absence or presence of Epidermal growth factor (EGF) receptors has been suggested as a valid approach for isolation of quiescent and activated type B cells, respectively (Beckervordersandforth et al., 2010; Codega et al., 2014). The use of EGF fluorescent ligands, combined with the CD24 (Calaora et al., 1996) and GFAP expression has also been employed to successfully isolate aNSCs and their progeny from the SEZ (Pastrana et al., 2009). Along the same line, Daynac et al. (2015) employed a triple staining based on the stem cell marker Lewis X (LeX/ssea 1) (Capela and Temple, 2002), EGFR and the neuroblast marker CD24 to isolate the different populations comprised within the lineage progression.

Adult SEZ does not only provide the olfactory bulb with new neurons. In this region, astrocytes and oligodendrocytes are also generated and migrate toward the corpus callosum, rostral migratory stream, white matter tracts of the striatum and the fimbria fornix, under both physiological conditions (Hack et al., 2005; Menn et al., 2006; Gonzalez-Perez and Alvarez-Buylla, 2011; Sohn et al., 2015; Tong et al., 2015), and demyelinating pathologies (Picard-Riera et al., 2002).

The other main area of neurogenesis in the adult brain is the SGZ, which produce dentate gyrus granular neurons involved in learning and memory (Shors et al., 2002; Zhao et al., 2008). This area, located at the interface of the granule cell layer and the hilus, harbors two types of neuronal progenitors. Type 1 progenitors display a radial glia-like morphology with a long process that crosses the entire granule cell layer and small processes horizontally oriented along the SGZ. These progenitors are characterized by the expression of GFAP, Nestin, and Sox2 (Seri et al., 2001; Fukuda et al., 2003; Garcia et al., 2004). Type 2 is the second population of progenitors that may arise from type 1 cells, have only short processes and lack GFAP expression. Type 1 and Type 2 progenitors give rise to intermediate progenitors, which in turn generate neuroblasts (Filippov et al., 2003; Fukuda et al., 2003). Subsequently, immature neurons migrate to the inner granule cell layer and differentiate into dentate granule cells in the hippocampus projecting axons through the hilus toward the CA3 region. These newborn neurons undergo a long process of maturation acquiring finally the electrophysiological and functional properties of their mature partners (reviewed by Ming and Song, 2011; Mongiat and Schinder, 2011; Kempermann et al., 2015).

The regulation of the cell fate specification of aNSCs is a critical point of discussion in which the balance between the role of intrinsic and extrinsic/environmental signaling is still not fully clarified. Recent studies have shown that within the SEZ internal programs would have a dominant role. According to these studies, SEZ seems to be highly regionalized, with neuronal progeny of distinct identity being generated at different areas along the dorsoventral and rostrocaudal axes (Hack et al., 2005; Merkle et al., 2007; Brill et al., 2009). Thus, aNSCs from the SEZ could be intrinsically specified on the basis of their location and they may display a limited potential of differentiation. Progenitors located along the SGZ on the other hand, seem to be more instructed by their microenvironment signals, regardless of the regional origin of the NSCs. For a detailed review of the existing knowledge of adult neurogenesis and the molecular mechanisms controlling it see (Zhao et al., 2008; Kriegstein and Alvarez-Buylla, 2009; Suh et al., 2009; Ming and Song, 2011).

In contrast to this more deterministic view, growing evidence suggest that SEZ progenitors exhibit a higher degree of plasticity, retaining the potential to generate larger diversity of neuronal subtypes (Sequerra et al., 2013; Fiorelli et al., 2015). Indeed, SHH signaling in the ventral SEZ is necessary for the generation of ventral-associated OB neurons (Ihrie et al., 2011; Merkle et al., 2014). Disruption of the SHH signaling redirects the ventral progenitors to a more dorsal cell fate, whereas overexpression of Smoothened leads to a “ventralization” of the dorsal SEZ (Ihrie et al., 2011). Likewise, exposure of postnatal and adult SEZ explants to an embryonic cortical environment, triggered an enhanced generation of glutamatergic neurons in addition to the predominant GABAergic subtypes (Sequerra et al., 2010). In addition, infusion of gamma amino butyric acid (GABA) into the OB induces the differentiation of newly generated cells into dopaminergic neurons (Akiba et al., 2009), suggesting a role for electrical activity in fate specification of those neurons. Thus, a parsimonious explanation for those data is that adult NSCs inherit genetic patterning of the embryonic RG (Merkle et al., 2007; Fuentealba et al., 2015), but this pattern is actively maintained by morphogenic signals and electrical activity in the adult SVZ. Changes in the external conditions could drive aNSCs into different neuronal lineages despite their embryonic origin and location in the SEZ.

### Live imaging of adult neural stem cells: applications and limitations

Understanding the mechanisms controlling the lineage progression of the adult neural stem cells (aNSCs) is critical for the future design of therapies against neurodegenerative diseases. However, the majority of the current knowledge regarding adult neurogenesis comes from population studies *in vivo*, leaving critical questions regarding the cell biology of the aNSCs unsolved. For example, how stem cells enact the decisions of self-renewal and differentiation on a single cell level is far from being understood. In fact, there is an ongoing discussion in the stem cell field whether such fate decisions are stochastic or deterministic in nature. Likewise, it is a matter of intense debate the concept of multipotency regarding aNSCs, i.e., whether a single aNSC is capable of differentiating into all the different cell types within their lineage or these distinct progenies constitute entirely separate lineages instead. Several studied reported that aNSCs exhibit multipotency when isolated *in vitro* from both rodents (Reynolds and Weiss, 1996; Gritti et al., 2002; Reynolds and Rietze, 2005; Vergano-Vera et al., 2009) and human brain (Sanai et al., 2004). However, this conclusion is mostly supported by experiments using the neurosphere assay, which has important caveats for assessing clonality (Singec et al., 2006; Pastrana et al., 2011). Moreover, as we will discuss in more details below, those studies also share the common feature of continuous exposure to mitogen factors, which can exert confounding effects on cell fate decisions (Doetsch et al., 2002; Costa et al., 2011). In contrast, recent evidences points to a more restricted outcome of the lineage progression, suggesting the bi-potency or even unipotency as the main hallmark of aNSCs in both the SEZ and the DG (Bonaguidi et al., 2011; Encinas et al., 2011; Ortega et al., 2013; Calzolari et al., 2015). That opens an interesting debate of which factors (as suggested by Ravin et al., 2008; Vergano-Vera et al., 2009) may re-direct the NSCs toward multipotency. Similarly, it is crucial to understand the mechanisms instructing aNSCs toward a specific cell fate *in vivo* and whether distinct populations of aNSCs contributing neurons or macroglial cells show different behaviors in lineage progression, such as cell cycle length, mode of cell division, and number of cell divisions (symmetric vs. asymmetric; Costa et al., 2011; Ortega et al., 2011, 2013; Ponti et al., 2013). Improved knowledge regarding all these questions will be determinant to understand how the generation of neurons, astrocytes, and oligodendrocytes is regulated in the adult germinative niches, allowing for the design of future therapeutic strategies focused on the replenishment of the cell loss due to pathological scenarios.

**Figure 2:**
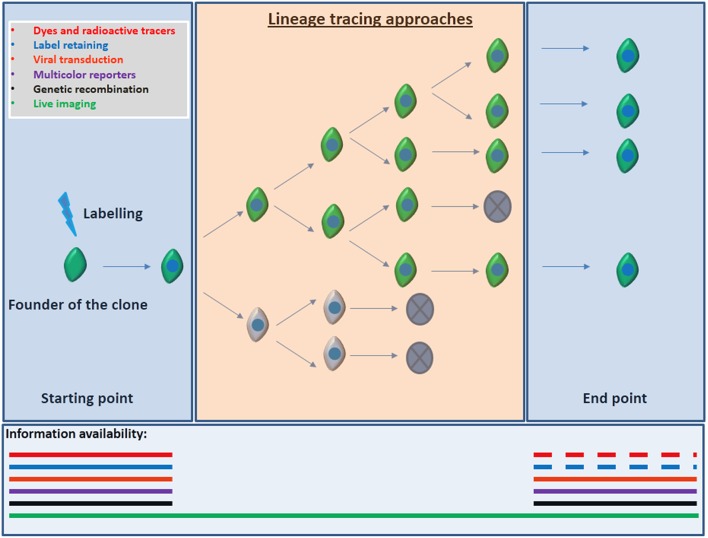
**Information available according to the lineage tracing approach employed**. Most of the lineage tracers implies a readout based on static end-point experiments. That implies that information regarding cell death, proliferation, differentiation, cell fate decisions, and migration inside or outside of the field of view of cell populations might be missing. Discontinuous segments of lines depict the lack of information that may also occur when using dyes or label retaining due to the dilution of the signal after several rounds of proliferation. Conversely, direct observation by live imaging allows for the visualization of each event from the founder of the clone, till the clone is formed improving the strength of the conclusions obtained from the experiment.

Lineage tracing, the identification, and monitoring of all the progeny of a single progenitor hold the key to elucidate these crucial interrogations. Ideal lineage tracing implies label retaining and specific marking of the founder of the clone (aNSC) and all its progeny without spreading the labeling to unrelated cells and without modifying the aNSC/progeny behavior. Several approaches have been used in order to perform lineage tracing: Dyes and radioactive tracers, viral transduction, transplantation, genetic recombination, and multicolor reporter constructs were used both *in vivo* and *in vitro*, in order to follow the NSCs and their progeny (reviewed by Kretzschmar and Watt, 2012). All these techniques enclose positive aspects but imply one main and critical drawback: the results and conclusions are based on still pictures along the lineage progression without comprising the whole sequence. This means that cell death, diffusion, dilution, spreading or low efficiency of the markers, among other important shortcomings could lead to incorrect conclusions Figure 2.

Although, constituting one of the oldest strategies for lineage tracing (Conklin, 1905; Sulston et al., 1983), live imaging followed by single cell tracking remains as the ideal method for the study of aNSCs lineage progression. Indeed, continuous observation of cell lineages over time allows studying the precise relations between a founder aNSC and its final clone, depicting in detail, with the adequate temporal resolution(Ravin et al., 2008; Eilken et al., 2009; Rieger et al., 2009), every intermediate step during the lineage progression. Using live imaging techniques *in vitro* we could predict crucial characteristics of the aNSCs cell biology (Costa et al., 2011; Ortega et al., 2011) later confirmed by BrdU-chasing or multicolor based clonal analysis *in vivo* (Ortega et al., 2013; Ponti et al., 2013; Calzolari et al., 2015). However, technical issues currently limit a comprehensive analysis of aNSCs lineage progression *in vivo* (see discussion below). Here, we will review some of the current techniques that have been developed to study the biology of the aNSCs by using live imaging in combination with single cell tracking. We will highlight the main advantages and limitations of both *in vitro* and *in vivo* approaches and discuss possible future directions toward a full understanding of aNSCs behavior, a crucial requirement in order to design future therapy strategies.

## Live imaging of aNSCs *in vitro*

Before describing in detail the present *in vitro* analysis that applied live imaging to the study of aNSCs, it is important to mention that the majority of the *in vitro* studies using aNSCs has been carried out by using the classical *in vitro* test for stem cell self-renewal, the neurosphere assay (Reynolds and Rietze, 2005). This method has been widely used to identify NSCs based on their capacity to evaluate self-renewal and differentiation at the single-cell level *in vitro*. However, as already discussed, several evidence indicate that the neurosphere assay may not be appropriate (Singec et al., 2006; Pastrana et al., 2011). Among several problems, is especially important the fact that clonality is not always guaranteed. Moreover, quiescent stem cells may not be detected by the neurosphere assay, in fact it was demonstrated that EGFR positive cells, i.e., activated type B cells and TAPs, are responsible for the generation of neurospheres (Doetsch et al., 2002; Pastrana et al., 2009). This clearly demonstrate that neurosphere assay does not reflect an accurate readout of the number of stem cells *in vivo*. In addition, this approach does not allow addressing the question of the stem cell mode of cell division due to its high cell density. Finally, the assay depends on the exposure of NSCs to mitogenic growth factors with their well-known confounding effects on cell fate decisions (Doetsch et al., 2002; Costa et al., 2011).

Therefore, live imaging of aNSCs required the development of new methods to perform successful and reliable single cell tracking. As we previously mentioned, the ideal situation would be the long-term imaging of single cells in whole living organisms, in real time and using several molecular readouts. However, the present available technology makes impossible the long-range immobilization of the organism as well as the access to the adult neurogenic areas (SEZ and SGZ) without the employment of highly invasive, and thus non-viable, approaches. In contrast, live imaging can be performed up to weeks *in vitro*, which allows for the analysis and manipulation of several molecular properties of aNSCs. Moreover, *in vitro* analysis provides crucial information regarding cell morphology, cell growth, migration, cell divisions patterns, cell cycle length, and cell fate decisions undergone by aNSCs (Costa et al., 2011).

There are several requirements to perform a good quality long-term live imaging experiment (reviewed in Schroeder, 2011). Live imaging followed by single cell tracking requires a complex balance between the imaging optimizations and the viability of the culture. Long or frequent exposition to excitation light, periodical changes in cell culture medium, adequate regulation of the gas composition of the incubator, among others, are technical aspects that may influence cell stress and toxicity. Moreover, the researcher has to establish carefully the settings of several peripheral components. For example, long-term live imaging requires adequate microscope systems. Bright-field and phase contrast are the systems most frequently used, in combination with motorized components that make possible a level of automation, indispensable for long term experiments (i.e., stages, shutters, and filters). Likewise the researcher has to count on incubation and permeable plates system able to keep the correct conditions of CO_2_ saturation and pH during the developing of the assay. Furthermore, the correct storage and subsequent processing of the results (i.e., images) requires expeditious hardware and software to deal properly with the huge amount of data that implies an experiment of live imaging. Finally, the accomplishment of the live imaging experiments depends often on the availability of specific fluorescent markers that allow the identification of the target population in real-time (Eilken et al., 2014).

Combining long term time-lapse video-microscopy with cultures seeded at clonal density Qian and colleagues provided important improvements in the knowledge of the behavior of embryonic NSCs. Culturing embryonic day (E)13 murine NSCs in medium supplemented with FGF2 they observed that the majority of the clones produced mainly neuroblast. These clones formed a heterogeneous population regarding their capacity of division, undergoing different rounds of amplifying divisions prior to the neuronal differentiation. Moreover, they were able to identify lineages containing both “neuroblast” and “glioblast” progenitors in these cultures (Qian et al., 1998). Lately, the use of time-lapse video-microscopy revealed that the production of neurons and glia followed a defined temporal pattern. Live imaging of different time points of embryonic NSCs isolated in culture indicated that neuroblast are generated first, whereas glia arise later. Furthermore, they confirmed that after the production of neuroblast and glioblast, NSCs in culture acquire features of postnatal progenitors, modifying their response to EGF (Qian et al., 2000). Recently, embryonic cultures (E12.5) in combination with time-lapse video-microscopy and computational processing and tracking have also been employed to elucidate behavioral differences between anterior (motor cortex-related) and posterior (visual cortex-related) neural progenitors (Winter et al., 2015). Single cell tracking of both populations indicate that posterior cortical progenitors exhibit higher speed in the cell cycle, increased cell size, and more motility than the anterior ones. As a consequence of the difference in the cell cycle length, the posterior progenitors generates larger clones that the anterior counterparts.

Clonal density cultures have been also used to image the lineage progression of rat or zebrafish embryonic retinal progenitor cells. Live imaging of single clones, controlling the composition of the extracellular environment and restricting completely the interaction between different clones demonstrated an unexpected degree of stochasticity in the progenitors regarding the decision between proliferation or differentiation into the different cell types located in the retina (Gomes et al., 2011; He et al., 2012). As we described previously for the aNSCs located in the SEZ, exits also an important line of research in retina suggesting that within the progenitor pool of the retina there is an important molecular heterogeneity. This heterogeneity would be determined by the presence of intrinsic genetic programs but also as a plastic response of progenitors to the influence of instructive extrinsic signals as Notch, Shh, GDF11 etc. (Cepko et al., 1996; Livesey and Cepko, 2001; Cayouette et al., 2006; Riesenberg et al., 2009).

Several modifications have been developed in order to improve the efficiency of the live imaging protocols. The use of extracellular matrix proteins in the coating defined a limited area where the cells are allowed to grow, creating then, a restricted environment that make the live imaging easier and more accurate. Combining this method with the manufacturing of a culture chamber that controls media and gas exchange over the microscope stage, Ravin and colleagues performed long term timelapse video-microscopy of E-14.5 NSCs. Manual reconstruction of the NSCs lineage trees revealed that under these culture conditions and in presence of FGF2, tripotent NSCs generate unipotent progenitors through an intermediate bipotent step, involving short phases of self-renewal within each independent stage. They also demonstrate that periodical passage of the progenitors in culture retrieve the tripotent capacity to the NSCs (Ravin et al., 2008).

Other significant improvements in the live imaging of NSCs involve techniques that allow the combination of time-lapse video-microscopy and single cell tracking in high density cultures instead of the previous clonal density. The application of these methods to the embryonic NSCs rendered the discovery of a new population of progenitors located in the marginal zone of the mouse cerebral cortex. This new embryonic niche harbors oligodendrogliogenic, astrogliogenic, and neurogenic progenitors as well as some bipotent NSCs (Costa et al., 2007). Another example was the descriptions of the role of Par-complex proteins in the lineage progression of embryonic NSCs (Costa et al., 2008). Par-complex proteins exhibit an apical gradual expression, being enriched in the ventricular radial glia and decreasing subsequently along the neurogenesis process. Time-lapse video-microscopy demonstrated that gain of function of Par3/Par6 proteins promoted self-renewal of ventricular progenitors whereas loss of function forced them to exit the cell cycle, regulating then, their mode of cell division (Costa et al., 2008). Finally, the use of high density cultures to further study the cell fate switch undergone by cortical NSCs during embryogenesis demonstrated that virtually all cortical progenitors generate neurons before giving rise to glia-committed progenitors (Costa et al., 2009).

Several researchers have tried to establish an automated method of tracking the lineage progression once the live imaging is accomplished. Different computational methods have been tested in murine embryonic NSCs cultures in order to monitor automatically the migration, proliferation, and cell growth of the clones along the timelapse video-microscopy, generating a lineage tree as a read out (Al-Kofahi et al., 2006; Winter et al., 2011). Likewise, Cohen and colleagues employed an automated algorithmic-information-theory-based method in order to obtain morphological and behavioral patterns. This data was used to predict with high levels of accuracy the mode of division (self-renewal vs. differentiation) and the cell fate decision (generated progeny) of rat retinal progenitors cells in culture (Cohen et al., 2010). However is important to mention here that, in contrast to retinal progenitors, most of the stem cells do not display an obvious characteristic morphology immediately after plating or along the culture. Although, some important improvements have been achieved in orders to reduce human validation in embryonic NSC cultures at clonal density (Winter et al., 2011, 2015), culture of NSCs requires often higher cell density, leading to a fast expansion and mixing of the clones generated depicting a much more complex scenario where current automated methods produce too many errors and requires of inevitable manual correction and interaction.

In contrast to their embryonic counterparts, aNSCs were mostly studied using neurosphere assay and other few culture systems conveying high concentrations of mitogenic factors (FGF2, EGF, or both) in culture medium. As we previously mentioned, these factors induce important alterations in the behavior of aNSCs (Doetsch et al., 2002; Costa et al., 2011) and may lead to inaccurate conclusions regarding the potency of these cells. Likewise, the use of live imaging on the study of the lineage progression of NSCs has long been restricted to the field of embryonic development, due to the technical limitations involved in singe-cell tracking in the neurosphere assay. To overcome these limitations, several laboratories developed adherent SEZ cultures that replicate some aspects of the SEZ *in vivo* (Lim and Alvarez-Buylla, 1999; Scheffler et al., 2005). Scheffler et al. reported an adherent culture, which involves expansion of stem/precursor cells in presence of EGF/FGF2 prior to differentiation (Scheffler et al., 2005). However, following this protocol, neural stem cells adopt tumorigenic properties (Walton et al., 2009), an effect that can be attributed to growth factor treatment (Kuhn et al., 1997; Doetsch et al., 2002). Lim and Alvarez-Buylla developed an interesting strategy of co-culturing adult SEZ cells with postnatal astroglia, which circumvent the necessity of growth factors for maintaining proliferation and neurogenesis. However, the requirement of such astroglial feeder introduces new uncontrollable variables: astroglia themselves secrete both growth factors and extracellular matrix molecules, likely affecting the intrinsic properties of adult neural stem cells; they introduce direct cell-contact mediated effects; they preclude analyse of direct effects of both intrinsic and extrinsic factor over SEZ cells, as astroglia feeder cells would also be affected; finally, due to the high cell density in such co-cultures single cell tracking becomes more cumbersome.

To circumvent these limitations, we recently developed a new method of culturing adult NSCs under adherent conditions and in absence of mitogenic factors (Costa et al., 2011; Ortega et al., 2011; see Table 1). Using this culture method, it was possible to monitor in real-time the lineage progression of aNSCs isolated from the lateral wall of the SEZ up to several days (Costa et al., 2011; Ponti et al., 2013). This technique allowed the direct observation of the intermediate steps between the progression from aNSCs to neurons: slow-dividing astroglia (type B cells) give rise to fast-dividing astroglia, which in turns generates TAPs. TAPs undergo subsequently several (up to 5) rounds of division giving rise to post-mitotic neuroblast in an orderly manner. These observations are in accordance with the sequence proposed by population-based studies *in vivo* (Doetsch et al., 1999a), but added two new pieces of information: (i) slow-dividing astroglia generate fast-dividing astroglial cells before TAPs and (ii) the number of cell divisions from aNSCs to the final neuronal progeny is limited. Moreover, this live-imaging system allowed the direct observation of asymmetric cell divisions within the lineage of aNSCs, providing a unique model for the study of NSC self-renewal. Interestingly, the asymmetry in the lineage is not observed in the first aNSC division, but rather at later points in the lineage. Finally, we could observe morphological changes characteristic of each cell type, such as the substantial cell growth prior to cell division of aNSCs, and the high motility of immature bipolar neuroblasts shortly after cell division.

**Table 1:** **Different methods employed for lineage tracing of aNSCs, highlighting some of their strongest advantages, and main weaknesses**.

	**Method**	**Strongest adventage**	**Main weakness**
Cells not monitored by live imaging	Retroviral vector	Allows to label dividing cells *in vivo* and *in vitro*.	The entire clone is not labeled. Cell death, migration away of a labeled cell, or immigration of a non-related labeled cells to the field of view are not discriminated.
	Genetic recombination and multicolor reporter systems	Allows to discriminate clonally while labeling high numbers of progenitors.	Cell death, migration away of a labeled cell, or immigration of a non-related labeled cells to the field of view are not discriminated.
	Classical neurosphere assay	Classical assay to evaluate NSCs hallmarks. (Self-renewal and differentiation capacity).	Does not ensure clonality, does not guarantee to monitor the slow dividing NSCs. Mitogen factors exerts confounding effects on the NSCs.
Cells monitored by live imaging	*In vitro* live imaging in presence of feeder layers/mitogen factors	Allows for continuous monitoring. Presence of niche factors.	Mitogen factors exerts confounding effects on the NSCs.
	Mitogen-free *in vitro* live imaging system	Allows for continuous monitoring. Perfect tool to evaluate the effect of each niche factor at a time. *In vivo* studies confirm their conclusions.	Absence of niche environment.
	Live imaging by brain slices	Allows for continuous monitoring. Preserves neurogenic niche.	Usually comprises high doses of serum in the preparation protocol. Restricted time of preservation of the neurogenic niche properties.
	Live imaging *in vivo*	Intact neurogenic niche in an intact brain environment.	Not yet developed for a single cell resolution.

This method was also applied to the study of other cell population generated in the adult SEZ, the oligodendrocytes (Menn et al., 2006; Gonzalez-Perez and Alvarez-Buylla, 2011). When the lateral and dorsal wall of the adult SEZ were cultured together, an increase in the number of oligodendroglial cells was observed, indicating, and enrichment of oligodendrogliogenic aNSCs in the dorsal aspect of the SEZ (Ortega et al., 2013). Timelapse video microscopy followed by single cell tracking revealed that aNSCs generating oligodendroglial progeny *in vitro* share several hallmarks (cell growth, slow cell cycle, etc.) with neurogenic aNSCs. Moreover, oligodendrogliogenic and the neurogenic aNSCs constitute strictly separate lineages, giving rise to either neurons and glia or oligodendrocytes and glia, respectively. Interestingly, we observed that the oligodendrogliogenic progeny is selectively activated by Wnt canonical signaling, known to be particularly prominent in the dorsal SEZ *in vivo* (Marinaro et al., 2012). Presence of Wnt recombinant proteins in the culture medium increased the rounds of amplifying divisions undergone by oligodendroglial progenitors by shortening their cell cycle. The expansion of the oligodendrogliogenic lineage induced by Wnt signaling within the SEZ has also been corroborated *in vivo* in both the adult (Ortega et al., 2013) and the postnatal (Azim et al., 2014) SEZ. It will be interesting to study the lineage progression of aNSCs when exposed to other extrinsic factors known to affect neurogenesis and oligodendrogenesis in the SEZ, such as BMP (ventral; Colak et al., 2008) and Shh (dorsal; Merkle et al., 2014; Tong et al., 2015).

One of the main criticisms regarding the study of cell behavior in culture is precisely that the progenitors are isolated from their “stem cell niche environment.” Therefore, important signals that regulate the aNSCs lineage progression could be missing and then, their behavior could be altered. Nevertheless, live-imaging of adherent SEZ cells under serum-free culture conditions allow to reproduce important steps in the lineage-progression of aNSCs and to describe new phenomena previously unnoticed (Costa et al., 2011; Ortega et al., 2011). In fact, results *in vivo* (Ponti et al., 2013) confirmed our observations *in vitro* (Costa et al., 2011) regarding aNSCs lineage progression and the number of cell divisions enacted in the lineage, indicating that important features of aNSCs are maintained in our culture system, despite the absence of niche signals. It is likely that SEZ cells isolated and observed in our experiments had already received environmental influences and the main merit of the system is not to use factor affecting the development of cell lineages.

Organotypic-slice cultures also constitute an interesting *ex-vivo* approach to study cell-divison and migration over short periods of time in embryonic (Noctor et al., 2004), posnatal (Raineteau et al., 2004; Namba et al., 2007; Yokose et al., 2011), and adult brain (Kamada et al., 2004). Combination of organotypic cultures, time-lapse microscopy, and two-photon microscopy uncovered interesting features of the motility of the SEZ-derived neuroblast (Nam et al., 2007; James et al., 2011). Contrary to expectations, it was shown that neuroblast migratory chains remain stable and immotile for longer periods. Moreover, brain slice cultures were also employed to demonstrate that adult NSCs are capable to regulate the local blood flow within the neurogenic niche by releasing ATP and vasodilating factors (Lacar et al., 2012). Organotypic cultures were also used to described alternatives models of oligodendrocytic-dependent myelination (Sobottka et al., 2011). However, to the best of our knowledge, aNSC lineage progression has not been comprehensively studied in organotypic culture. One important challenge in this system is to maintain healthy slices over long periods of time. In fact, neural precursors have a limited capacity to differentiate into neurons and the structural integrity of the neurogenic niche seems to be compromised during the culture period (Namba et al., 2007). One possible explanation to these problems is the high concentration of serum required to keep slices alive. Lastly, it is important to consider that the different steps observed in the aNSC lineage start in the SEZ, progress over the RMS and end in the olfactory bulb. Therefore, the slice must keep all these structures intact and the live-imaging set up to follow all those steps must be able to image cells at long distances. Nevertheless, we believe that organotypic cultures could be improved, allowing longer survival periods and more physiologic neurogenic rates, and then used to monitor aNSC behavior within the SEZ.

## Live imaging of aNSCs *in vivo*

As discussed in the previous sections, one potential pitfall of studying aNSCs *in vitro* could be the absence of the niche. In fact, organotypic-slice cultures may keep some local relations, but do not maintain long-distance axonal influences involved in the control of aNSCs behavior (Tong et al., 2014). Thus, the perfect situation would be to be able to image aNSCs within their niche under physiological conditions *in vivo*. However, imaging techniques so far available to image the brain *in vivo* do not allow the study of aNSCs in a single-cell manner.

Up to date, there are three main imaging techniques used to image NSCs and neurogenesis in the adult live brain: Magnetic resonance imaging/spectroscopy (MRI/MRS); Positron emission tomography (PET); and optical imaging (fluorescence and bioluminiscence). However, as discussed below, each of these techniques has limitations that preclude their use to study the behavior of single cells in the live brain.

MRI offers a relatively high morphological resolution in deep regions of the brain (78 × 78 × 370 μm) (Weber et al., 2006). However, this resolution does not allow studying single cells. In fact, MRI has been mostly used to evaluate volumetric modifications in regions where neurogenesis occurs under physiological or pathological conditions and to correlate these changes with possible alterations in the rate of neurogenesis (Couillard-Despres and Aigner, 2011).

To identify specific cell population using MRI, some researches have used iron oxide particle labeling of neural stem and precursors cells *in vitro* and then transplanting labeled cells into aNSC niches *in vivo* (Hoehn et al., 2002). Using this technique, it has been possible to image transplanted cells over several weeks in the host brains and follow their migration. However, *in vitro* labeling of cells may lead to changes in several properties of the cells, hampering the enthusiasm regarding the use of such technique to study the physiological behavior of aNSCs.

In order to overcome this limitation, some groups have attempted to label endogenous neural stem/precursos cells *in vivo* through the injection of iron particles directly into the lateral ventricles or in the subventricular zone. These works have shown that particles can be uptaken by migrating neuroblasts, allowing the observation of their migration toward the olfactory bulb by MRI. Thus, particle-labeling of cells is a promising technique to study adult neurogenesis *in vivo* by non-invasive imaging (Panizzo et al., 2009; Granot et al., 2011; Iordanova and Ahrens, 2012).

Nevertheless, several issues must be taken into consideration in these studies: (i) uptake of particles by SVZ cells is unspecific, in other words, it is not possible to distinguish which type of cell (ependymal, type B, C, or A cell) is being observed; (ii) injection of iron particles *in vivo* may lead to cell toxicity (Crabbe et al., 2010; Vreys et al., 2010); and (iii) iron particles may be uptaken by other cells following death of the primary-carrier cells, which could cause a great confusion in the analysis of data. Together, these technical issues embody serious limitations to the use of MRI to study aNSC behavior *in vivo*.

An elegant way to identify specific cell populations has been proposed by Manganas et al. (2007). They used MRS to identify possible “metabolic biomarkers” for different cell types in the adult hippocampus. For example, they reported a MRS peak, detected at 1.28 ppm (parts per million), which seemed to correlate with the presence of neural stem cells (Manganas et al., 2007). The authors reported an increase in the 1.28-ppm biomarker in the dentate gyrus of rodents following electric convulsive shock, which could be interpreted as a sign for increased endogenous neurogenesis. Conversely, they described a reduction of the 1.28-ppm biomarker in the hippocampus of adult humans, as compared to preadolescents and adolescents. Based on these findings, they concluded that the 1.28-ppm biomarker could be applied to track and analyze endogenous NSCs *in vivo* (Manganas et al., 2007). However, Ramm et al. (2009) demonstrated that the 1.28-ppm peak was also evident in mesenchymal stem cells and in non-stem cell lines. Notably, this peak could only be observed in neural progenitor cells cultured under conditions favoring growth arrest or apoptosis, suggesting that such peak could be caused by the appearance of mobile lipid droplets during apoptosis (Ramm et al., 2009). Therefore, the validity of the 1.28-ppm biomarker as a measure for NSCs and neurogenesis requires additional evidence.

PET has also been used to study neurogenesis in the rodent adult brain (Rueger et al., 2010). Using the ^18^F-labeled 3′-deoxy-3′-fluorothymidine (FLT), a thymidine analog incorporated to newly synthesized DNA molecules, they could observe an increase in the PET signal both in the hippocampus and subventricular regions in the ipsilateral hemisphere subjected to ischaemic lesion (Rueger et al., 2010). However, although such increase has been interpreted as readout for augmented neurogenesis, the signal intensity observed in the hippocampus and SVZ seemed very similar, even though neurogenesis in the SVZ is 10-100 times higher. One possible explanation for this discrepancy could be the low resolution of PET in comparison to MRI.

Needless to say, incorporation of ^18^F-FLT is unspecific and, consequently, does not allow the identification of cell types imaged by PET. Thus, the use this technique to study endogenous NSCs and neurogenesis *in vivo* remains a forthcoming alternative.

Compared to MRI/MRS and PET, optical imaging based in fluorescence or bioluminescence is a cheaper and more versatile method to study cells *in vivo*. Using any microscope equipped for epifluorescence acquisition, for instance, it is possible to exploit all the advantages of transgenic animals expressing fluorescent proteins controlled by specific promoters or virally-transduced markers, prompting the identification of specific cell types, and achieve resolution sufficient for morphological studies at cellular and subcellular levels.

Although, light penetration in the live brain is an important drawback for fluorescence live imaging in the SVZ and hippocampus, the advent of multi-photon confocal microscopy has significantly improved the quality of fluorescence imaging in deep regions of the brain (Lendvai et al., 2000; Grutzendler et al., 2002; Trachtenberg et al., 2002). In a seminal work, Mizrahi et al. used two-photon microscopy to image the olfactory bulb of transgenic mice expressing GFP in juxtaglomerular neurons (JGNs), a population that undergoes adult neurogenesis, over periods of up to 3 months, *in vivo*. Based on GFP identification, they could perform time-lapse analysis and demonstrate that JGNs in the olfactory bulb have a turnover rate of about 3% per month (Mizrahi et al., 2006). Recently, this imaging technique has been combined to treatment with calcium indicators and used to study the functional integration of newly generated neurons in the adult rodent olfactory bulb (Kovalchuk et al., 2015).

However, neurogenic niches in the SVZ and hippocampal DG exist in much deeper brain regions as compared to the regions studied in the above cited work (Lendvai et al., 2000; Grutzendler et al., 2002; Trachtenberg et al., 2002; Mizrahi et al., 2006), hampering the use of two-photon microscopy to study endogenous aNSCs.

Recently, however, the group of David W. Tank has developed a preparation to image adult hippocampal cells in wake animals (Dombeck et al., 2010). In order to image neuron activity in CA1 during navigation, the authors removed the overlaying cortical tissue by aspiration and placed a stainless cannula that functioned as a window to CA1 of adult rats. Two-photon time-lapse microscopy of CA1 neurons labeled with the calcium indicator GCaMP3 during navigation in a virtual reality system allowed the identification of place cells and the correlation between the location of their place fields in the virtual environment and their anatomical location in the local circuit (Dombeck et al., 2010).

These data indicate that deeper regions of the brain can be imaged following removal of covering structures. However, to the best of our knowledge, this has not yet been tested to image aNSCs either in the DG or SVZ. Furthermore, it is important to keep in mind that surgical procedures to remove brain tissue may have critical influences in aNSC behavior and neurogenesis. In fact, several models have shown changes in the rate of proliferation within neurogenic niches following injury (Arvidsson et al., 2002; Collin et al., 2005; Aponso et al., 2008; Saha et al., 2013).

## Imaging of local progenitors in the cerebral cortex

It has also been proposed that cells with stem/progenitor potential reside within the cortical parenchyma and could be activated under specific condition, such as injuries. Because these cells are located in superficial regions of the brain, it is possible to study their behavior by two-photon time-lapse microscopy.

The cerebral cortex layer 1, the most superficial layer of the cerebral cortex, for example, harbors a population of progenitors capable of generating neurons and glial cells during development (Costa et al., 2007; Breunig et al., 2012). Although, neurogenesis from layer 1 progenitors seems to be restricted to embryonic and postnatal stages under physiological conditions, these progenitors may resume proliferation after ischemic injury in adult animals and generate neurons (Ohira et al., 2010). Given the matchless superficial location of layer 1 progenitors, they are good candidates to imaging by two-photon microscopy.

Another interesting cell population for imaging in the adult brain is the parenchymal astroglia. Following traumatic and ischemic injury, astroglial cells proliferate, and acquire several hallmarks of neural stem cells (Buffo et al., 2008; Sirko et al., 2013), leading to the suggestion that parenchymal astroglia could be an interesting source for neuronal replacement under different neurological conditions (Robel et al., 2011; Chouchane and Costa, 2012).

In a recent work, Bardehle et al. (2013) used *in vivo* two-photon laser-scanning microscopy to follow the response of GFP-labeled astrocytes in the adult mouse cerebral cortex over several weeks after acute injury. The authors described a selective proliferation of juxtavascular astrocytes after the introduction of a lesion, indicating that astrocyte recruitment after injury relies solely on proliferation in a specific niche (Bardehle et al., 2013). If these proliferating astrocytes are the cells acquiring neural stem cell properties *in vitro*, two-photon time-lapse imaging of these cells might bring some insights about aNSC behavior *in vivo*.

## Conclusion

Live-imaging of isolated SVZ cells *in vitro* has contribute to deepen our knowledge about the lineage-progression of adult NSCs. However, *in vivo* imaging systems still lack resolution to resolve single-cell lineages. Future studies should address the contribution of individual NSCs to different neuronal lineages and the possible influence of signaling molecules in this process, both *in vitro* and *in vivo*. Long-term imaging *in vivo*, however, awaits future developments in microscopy that will permit the observation of cells generated in the SVZ and the following up of these cells through the RMS and in the OB. Alternatively, short time live-imaging of aNSCs within the SEZ *in vivo* combined with multicolored fate-mapping could help to describe how the progeny of single NSCs, identified in the olfactory bulb by multicolor codes, is generated.

## Author contributions

FO organized and coordinated the preparation of the present review. FO and MC contributed to the writing of the manuscript.

## Funding

FO was supported by research grants MICINN (BFU2011-24743), Consolider-Ingenio 2010 “Spanish Ion Channel Initiative” (CSD2008-00005), MEC (BFU2014-53654-P), and BRADE-CM (S2013/ICE-2958) and the Ramon y Cajal Program of the Spanish Ministry of Economy and competitiveness (MEC) (RYC-2013-13290). MC is supported by CNPq, CAPES, and Fundação de Amparo à Pesquisa do Rio de Grande do Norte (FAPERN).

### Conflict of interest statement

The authors declare that the research was conducted in the absence of any commercial or financial relationships that could be construed as a potential conflict of interest. The reviewer CV and handling Editor declared their shared affiliation, and the handling Editor states that the process nevertheless met the standards of a fair and objective review.
